# Effects of a 12-Week Hatha Yoga Intervention on Cardiorespiratory Endurance, Muscular Strength and Endurance, and Flexibility in Hong Kong Chinese Adults: A Controlled Clinical Trial

**DOI:** 10.1155/2015/958727

**Published:** 2015-06-08

**Authors:** Caren Lau, Ruby Yu, Jean Woo

**Affiliations:** Department of Medicine and Therapeutics, The Chinese University of Hong Kong, Sha Tin, Hong Kong

## Abstract

*Objective*. To examine the effects of a 12-week Hatha yoga intervention on cardiorespiratory endurance, muscular strength and endurance, and flexibility in Chinese adults.* Methods*. 173 adults (aged 52.0 ± 7.5 years) were assigned to either the yoga intervention group (*n* = 87) or the waitlist control group (*n* = 86). 19 dropped out from the study. Primary outcomes were changes in cardiorespiratory endurance (resting heart rate (HR) and maximal oxygen uptake (VO_2max_)), muscular strength and endurance (curl-up and push-up tests), and lower back and hamstring flexibility (the modified back-saver sit-and-reach (MBS) test).* Results*. Compared to controls, the yoga group achieved significant improvements in VO_2max_ (*P* < 0.01), curl-up (*P* < 0.05) and push-up (*P* < 0.001) tests, and the MBS left and right leg tests (both *P* < 0.001) in both genders. Significant change was also found for resting HR between groups in women (*P* < 0.05) but not in men. Further analysis comparing participants between younger and older subgroups yielded similar findings, except that the older participants in the yoga group failed to improve resting HR or the curl-up test versus control. Adherence (89%) and attendance (94%) were high. No serious adverse events occurred.* Conclusion*. A 12-week Hatha yoga intervention has favorable effects on cardiorespiratory endurance, muscular strength and endurance, and flexibility in Chinese adults.

## 1. Introduction

The health benefits of enhancing physical fitness (i.e., cardiorespiratory fitness (CRF), muscular fitness, and flexibility) have become well established during the past decades. Higher levels of CRF and muscular fitness are associated with significantly lower risk of developing metabolic syndrome [1, 2] and all-cause and cardiovascular mortality [3–7]. Although physical fitness declines as part of the physiological changes with age [8–10], the rate of decrease and possible reversibility might be amendable by intervention. Accumulating evidence indicates that an active lifestyle helps preserve CRF [11]. Furthermore, there is evidence around the benefits of aerobic and resistance exercises to improve CRF, muscular fitness, and health-related factors [12–14], thus highlighting the importance of intervention modalities. However, a large proportion of adults are much less active than desired [15–17], where the major barriers include physical limitations, low self-efficacy, or simple aversion to exercise [18–20].

Increasing evidence suggests that complementary and alternative approaches that encourage increased physical activity and reduce sedentary behaviours might confer health benefits. Originated in India, yoga has become increasingly popular in western countries [21] as a means of exercise primarily using gentle static stretching postures with minimal physical exertion and conscious breathing to promote flexibility and relaxation. Of the various branches of yoga (such as Hindu, Hatha, Raja, and Mantra), Hatha yoga is perhaps the most widely practiced, which consists of elements of physical postures, conscious breathing, and meditation [22]. Hatha yoga appears safe and easy to learn and does not require any complicated or expensive equipment or specific training venue and thus could be suggested as an alternative form of exercise associated with high exercise adherence [23].

In addition to the beneficial effects on flexibility and relaxation, some yoga postures may achieve the recommended level of intensity for cardiovascular fitness [24, 25]. A growing number of research studies have shown that Hatha yoga can lead to improvements in CRF and muscular strength [26–29]. A recent review provides preliminary evidence of improvements in strength, balance, aerobic fitness, and self-rated health after yoga practice [30]. These benefits may be particularly important for people who are unable or unwilling to participate regularly in aerobic or resistance exercises. However, studies on the effects of yoga on CRF and muscular fitness have been few and have involved a small number of participants. The effects of yoga on physical fitness in Chinese adults have not been reported.

To fill these knowledge gaps, we developed a 12-week Hatha yoga intervention in the community to evaluate the effects of the intervention on cardiorespiratory endurance, muscular strength and endurance, and flexibility in a two-arm 12-week, prospective, nonblinded controlled trial enrolling Hong Kong Chinese adults. We also documented intervention adherence, attendance, and acceptability.

## 2. Materials and Methods

### 2.1. Participants

173 Chinese men and women aged 18 and above were recruited for the study between May 2010 and January 2012. Recruitment was done by newspaper advertising, by placing notices in community centers, and by Internet publicity (including emails, advertisement, discussion forums, and website). Participants were volunteers, and the aim was to recruit a stratified sample so that similar proportions of males and females were obtained. An enrolment form was used for the screening purposes. Chinese individuals aged 18 and older, able to communicate in Cantonese, and physically and mentally capable of practicing yoga safely were included. Those who had severe medical conditions which limit their abilities to complete the whole course of treatment were excluded. In addition, those who were concurrently participating in yoga, qigong, meditation or other research studies were excluded.

### 2.2. Procedure

**Figure 1:**
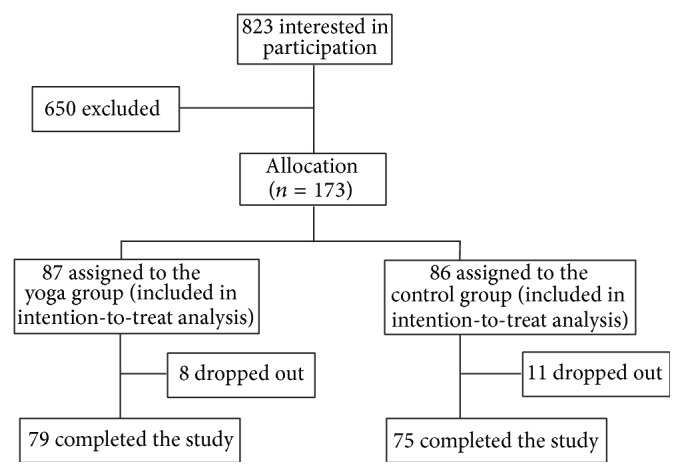
Recruitment of participants.

The study had adopted a prospective two-arm nonblinded controlled design. Each participant was individually assessed and they were grouped as 87 for yoga and 86 for control groups (Figure 1). To ensure that the proportions of male/female in the yoga and control groups would be similar and comparable for subgroup analysis, quota sampling was adopted, with gender used as quota control. Demographics, medication use, lifestyle factors, health-related quality of life (HRQoL), body measurements, and a battery of health-related physical fitness measures (described below) were taken before and after the 12-week protocol. Baseline assessments were performed between July 2010 and January 2012. Follow-up assessments were performed between October 2010 and May 2012. The study was conducted within a university-affiliated hospital (Prince of Wales Hospital, Sha Tin, New Territories, Hong Kong). All eligible participants participated voluntarily and their written informed consent was obtained prior to the study. The study was conducted as per the tenets of the Declaration of Helsinki with approval from the Joint Chinese University of Hong Kong-New Territories East Cluster Clinical Research Ethics Committee (registration number: CRE-2010.115; date of approval: 27 April 2010). The trial has been retrospectively registered in the Australian New Zealand Clinical Trials Registry (registration number: ACTRN12613000816752). The authors confirm that all ongoing and related trials for this intervention are registered.

### 2.3. Yoga Training

The yoga group participants were invited to attend a yoga training program consisting of 12 weekly 60-minute sessions, which were conducted by an Experienced Registered Yoga Teacher (E-RYT) (Yoga Alliance) with more than four years of Hatha yoga instructing experience. Throughout the 12 training sessions, participants were arranged in groups of seven to ten and were taught the breathing technique and 57 yogic poses commonly taught in community fitness centres including (1) standing poses which include Chair Pose (*Utkatasana*) and its variation (i.e., Chair with Torso Twist), Extended Hand-Toe Pose (*Utthita Hasta Padangusthasana*), Extended Side Angle Pose (*Utthita Parsvakonasana*), Half Moon (*Ardha Chandrasana*), Modified Half Moon, Intense Side Stretch (*Parsvottanasana*), King of the Dancers Pose (*Natarajasana*), Lunge, Mountain Pose (*Tadasana*), Revolved Side Angle Pose (*Parivrtta Parsvakonasana*), Revolved Lunge, Revolved Triangle Pose (*Parivrtta Trikonasana*), Squat-Sitting-Down Pose (*Malasana*), Standing Forward Bend (*Uttanasana*), Tree Pose (*Vrkshasana*), Triangle Pose (*Trikonasana*), Warrior I (*Virabhadrasana I*), Warrior II (*Virabhadrasana II*), Warrior III (*Virabhadrasana III*), and Wide-Stance Forward Bend (*Prasarita Padottanasana*) and its variation (i.e., Wide-Stance Forward Bend with Torso Twist), (2) sitting poses which include Boat Pose (*Navasana*), Bound Angle Pose (*Baddha Konasana*), Cow-Face Pose (*Gomukhasana*), Half Load of the Fishes Pose (*Ardha Matsyendrasana*), Head-to-Knee Pose (*Janu Shirshasana*), Marichi I (*Marichyasana I*), Pigeon Pose (*Rajakapotasana*), Revolved Head-to-Knee Pose (*Parivrtta Janu Shirshasana*), Seated Forward Bend (*Paschimottanasana*), and Seated Wide-Angle Pose (*Upavistha Konasana*), (3) kneeling poses which include Camel Pose (*Ushtrasana*), Child's Pose (*Balasana*), Gate Pose (*Parighasana*), and One-Legged Royal Pigeon and Folded Forward (*Rajakapotasana*), (4) supine which include Apana Pose (*Apanasana*), Belly Twist (*Jathara Parivartanasana*), Bridge Pose (*Setu Bandhasana*) and its variation (i.e., Bridge with One Leg Lift), Corpse Pose (*Shavasana*), Preparation exercise for Plow Pose, Plow Pose (*Halasana*), Reclining Bound Angle Pose (*Supta Baddha Konasana*), Side-Reclining Leg Lift (*Anantasana*), and Supported Shoulder Stand (*Salamba Sarvangasana*), (5) prone poses which include Bow (*Dhanurasana*), Cobra (*Bhujangasana*), Locust (*Shalabhasana*), and Upward Facing Dog (*Urdhva Mukha Svanasana*), and (6) arm support poses which include Cat Cow (*Durga Go*) and its variation (i.e., Cat Cow with One Leg Lift), Four-Footed Table Top Pose (*Chatus Pada Pitham*), Plank (*Utthita Hasta Padangusthasana*), Table Top Exercise, Side Plank (*Vasisthasana*), and Upward Plank (*Purvottanasna*). Participants were also encouraged to practice yoga at home between classes (with handouts of yogic poses), and self-practice log sheets were used. Apart from the yoga program, participants in the yoga group were advised to maintain their routine activities and not to begin other exercise or mind-body program during the course of the study.

### 2.4. Control Group

The control group participants were requested to maintain their routine activities and not to begin any exercise, yoga, or mind-body program during the course of the study. To ensure adherence of the protocol, the control group participants also received the yoga program after the end of the study period.

### 2.5. Measurements

#### 2.5.1. Cardiorespiratory Endurance

Resting heart rate (HR) was measured following a seated ten-minute rest period. The HR was detected by electronic device (Polar Electro, Finland). Maximal oxygen uptake (VO_2max_) was assessed with a maximal treadmill exercise test according to the Bruce protocol [31, 32]. Participants were instructed to abstain from any strenuous exercise on the day before testing. Each participant was connected to a calibrated respiratory gas analyzer (Fitmate, COSMED Srl, Italy) for gas analysis using a face mask. The respiratory gas analyzer was calibrated before each test. Treadmill speed was set initially at 1.7 miles per hour (mph) and 10% grade. Every three minutes, the speed (and grade) increased to 2.5 (12%), 3.4 (14%), 4.2 (16%), 5.0 (18%), and finally 5.5 (20%). Participants were verbally encouraged to reach their maximum. The test was terminated when the participant reached peak VO_2_ [33, 34] or showed any symptoms that indicated termination of exercise based on the guidelines of the American College of Sports Medicine [32].

#### 2.5.2. Muscular Strength and Endurance

The Canadian Standardized Test of Fitness (push-up and curl-up tests) was conducted to measure the muscular endurance of upper body muscles and the abdominal muscle groups, respectively. The test procedures for the measurements were according to the descriptions of Canadian Society for Exercise Physiology (CSEP) [32].

*Push-Up Test.* The participant started with the standard “down” position (the male participant was instructed to have hands pointing forward and under the shoulder, back straight, head up, using the toes as the pivotal point, whilst the female participant was instructed to have both legs together, lower leg in contact with mat with ankle plantar-flexed, back straight, hands shoulder width apart, head up, using the knees as the pivotal point). The participant raised the body by straightening the elbows and returning to the “down” position until the chin touches the mat and the abdomen should not touch the mat. The maximal number of push-ups performed consecutively without rest was counted as the score. The test was terminated when the participant strained forcibly or was unable to maintain the appropriate technique within two repetitions. 

*Curl-Up Test.* The participant was instructed to perform a supine position on a mat with the knees at 90 degrees and place the hands on the thighs and curl up until the hands reach the knee caps. Shoes remain on during the test. The mobile metronome [35] was set to 50 beats per minute and the participant did slow, controlled curl ups to lift the shoulder blades off the mat (truck made 30-degree angle with the mat) in time with the metronome at a rate of 25 per minute. The test was conducted for one minute. The lower back had to be kept flattened before curling up. The participant was encouraged to perform as many curl-ups as possible without pausing, to a maximum of 25 [32].

#### 2.5.3. Flexibility

The modified back-saver sit-and-reach (MBS) test was used to test lower back and hamstring flexibility [36]. The participant was requested to sit on a 30 cm high bench with one leg extended and resting on the bench, whilst the foot of the other leg was placed on the floor. A meter rule was placed on the bench between the legs and the heel of the extended leg was in line with the 50 cm level on the meter rule. The participant was required to stretch both arms out in front of the body with hands held together and fingers pointing toward the extended leg and was reminded to reach as far forward as possible without causing pain in the extended leg. The maximum distance that the tips of the middle fingers of both hands reached, as read from the meter rule, was indicated as the score of lower back and hamstring flexibility. Three trials were conducted with each leg and the maximum score for each leg (to the nearest mm) was recorded and entered for analysis [37]. The MBS test was a comparatively better test to measure the lower back and hamstring flexibility than other protocols as its similarity of criterion-related validity in women but it had better criterion-related validity in men, more practical, as it required minimal preparation time and equipment. It also eliminated excessive posterior compression of the vertebral disk when performing a single leg reach [36].

#### 2.5.4. Other Covariates

Information on a number of covariates was also collected. Age, gender, marital status, education level, occupation, medication use, smoking, alcohol intake, physical activity level (assessed with the International Physical Activity Questionnaire, IPAQ [38, 39]), HRQoL (assessed with the Medical Outcomes Study (MOS) 36-item Short-Form Health Survey, SF-36 [40–42]), systolic blood pressure (SBP), diastolic blood pressure (DBP), and body mass index (BMI) were obtained by questionnaire interviews and measurements.

### 2.6. Data Processing and Analysis

We analysed the outcome variables in the intention-to-treat (ITT) population consisting of participants who completed the study protocol and those who completed the baseline assessment but dropped out from the study afterward. We used carry-forward imputation to estimate the missing follow-up data in ITT population. Continuous and categorical variables were summarized as mean (SD) or by counts and percentages. We checked the homogeneity of the yoga and control groups with independent *t*-test and Mann-Whitney *U* test for continuous variables and Chi-square (*χ* ^2^) test for categorical variables. Relationships between baseline characteristics and the outcome variables were determined with correlations. By incorporating findings from homogeneity check and correlations, possible covariates were identified. We tested the mean difference of each outcome variable between the yoga and control groups with analysis of variance (ANOVA) or analysis of covariance (ANCOVA) when possible covariates were identified. To assess the magnitude and direction of the effect of the yoga intervention relative to the control condition for each outcome variable, effect sizes were computed, where the value of partial eta-squared (*η* ^2^) is represented as very small (<0.01), small (0.01–0.05), medium (0.06–0.13), and large (≥0.14). Covariates included BMI and DBP as well as HRQoL role emotional domain score and mental health component score. Men and women were initially analysed separately in statistical tests and the analyses were repeated in younger and older participants (below or above the median of the age distribution, i.e., 53.0 years) with both genders combined due to the small sample size of each subgroup. All statistical tests were two-tailed and the acceptance level of statistical significance (*P* value) in overall analysis was 0.05 or less. All statistical analyses were carried out using Windows-based Statistical Package for the Social Sciences version 21.0 software (SPSS, Chicago, IL, USA).

## 3. Results

### 3.1. Baseline Characteristics

A total of 823 individuals were screened for eligibility and 66 were excluded due to health conditions. 39 prospective participants declined participation (reasons included their failure to make themselves available for attending the assessments or yoga classes) and 34 could not be contacted after multiple call attempts. Among the 684 prospective participants, 173 men (*n* = 64) and women (*n* = 109) were recruited and assigned to either the yoga group (*n* = 87) or the control group (*n* = 86), of which 19 dropped out from the study.

The baseline characteristics of participants are shown in Table 1. The mean age was 52.0 ± 7.5 (31.0–71.0) years. Most of the participants were married (81.5%) and had secondary education (93.6%). About half engaged in full-time jobs (53.8%). Since the two genders differed significantly in various baseline characteristics and the outcome measures, subsequent analyses were undertaken separately for men and women.

In comparing the yoga and the control group, in both genders, there were no significant differences in baseline characteristics except that, in men, the yoga group had lower flexibility (MBS leg and right leg tests, both *P* < 0.01), whereas in women, the yoga group had lower HRQoL role emotional domain score and BMI (both *P* < 0.05, Table 2).

### 3.2. Effects of Intervention on Outcome Measures

In men, the yoga group achieved greater increase in VO_2max_ than the control group (*P* < 0.01). Significant improvements were also found for muscular strength and endurance in the yoga group compared with the control group (curl-up test *P* < 0.05, push-up test *P* < 0.001). Lower back and hamstring flexibility increased significantly in the yoga group but not in the control group (MBS both left and right leg tests *P* < 0.001). No significant differences were found for resting HR between groups (*P* = 0.086). In women, the yoga group also achieved greater increase in VO_2max_ (*P* < 0.01), the curl-up (*P* < 0.05) and push-up (*P* < 0.001) tests as well as the MBS left and right leg tests (both *P* < 0.001) than the control group. Significant improvement was also found for resting HR in the yoga group compared to the control group (*P* < 0.05, Table 3).

The analyses were repeated in younger and older participants, with both genders combined. In younger participants, the yoga group achieved greater improvements in resting HR (*P* < 0.05), VO_2max_ (*P* < 0.01), the curl-up (*P* < 0.01), and push-up (*P* < 0.001) tests as well as the MBS left and right leg tests (both *P* < 0.001) than the control group. In older participants, the yoga group also achieved greater improvements in VO_2max_ (*P* < 0.01), the push-up test (*P* < 0.001), and the MBS left and right leg tests (both *P* < 0.001) than the control group. However, no significant differences were found for resting HR (*P* = 0.128) and the curl-up test (*P* = 0.103) between groups (Table 4).

### 3.3. Adherence, Attendance, and Acceptability

The overall adherence rate was 89% (yoga group: 92%, control group: 87%) and the mean attendance rate of 12 sessions was 94%. According to the log records for practice at home, 99% of the yoga participants practiced yoga at home for an average of about 165 minutes/week (23 minutes/day). Qualitative feedback indicated that the intervention was well received, with most of the participants reporting improvements in health after yoga participation. While some of the participants reported some difficulties with the yoga postures at the beginning of the intervention, no unanticipated adverse events were reported.

## 4. Discussion

The present study was conducted to examine the effects of a 12-week Hatha yoga on cardiorespiratory endurance, muscular strength and endurance, and lower back and hamstring flexibility in Hong Kong Chinese adults. Our results demonstrated significant improvements in VO_2max_, muscular strength, and flexibility in both men and women who practiced yoga compared to the control group. The yoga group also showed a reduction in resting HR compared to the control group in women. Further analysis comparing participants between younger and older subgroups yielded similar findings, except for the older participants in the yoga group, who failed to improve the resting HR or the curl-up test versus the control group.

Studies investigating the effects of yoga on cardiorespiratory endurance are limited. Sivasankaran et al. [43] showed that the resting HR was significantly reduced by 9 beats/minute after a 6-week program of yoga and meditation in people with and without coronary artery disease. Another study on the effects of yoga on resting HR and blood pressure in middle-aged adults was reported by Devasena and Narhare [44] who found that a 6-month yoga program (with 1-hour daily practice session) could significantly reduce resting HR over the course of the intervention. However, these two studies have been performed without a comparison group. Using a nonblinded controlled study design, the present study showed no benefit to men with regard to resting HR, but a modest effect was observed in reducing resting HR in women. The reduction observed in women may be mediated by the training-induced reduction of respiratory rate and improvement in the baroreflex sensitivity [45–47], attributed to the relaxation and breathing techniques adopted in yoga training [48]. The lack of significant improvement in resting HR in men does not necessarily imply that the yoga intervention is not beneficial to cardiorespiratory endurance but may relate to the relatively lower resting HR at baseline in men (67.17 beats/minute) compared with women (69.88 beats/minute), so that the effect of yoga regarding this measure in men was less pronounced than that in women. Our findings also demonstrated a significant improvement in resting HR in the younger but not in the older participants. This is in contrast to a previous yoga intervention conducted by Bowman et al. [49] who has reported a significant reduction in resting HR following a 6-week yoga program in elderly person aged 62–81 years. This disparity between studies could be attributed to many factors, including differences between intervention and population characteristics. Respiratory rate is a possible confounding factor. However, we have no such measurement in the study.

In addition to improving resting HR, the yoga intervention increased VO_2max_ in both men and women compared with the control group. When comparing participants between younger and older subgroups, the yoga intervention was similarly effective in increasing VO_2max_ in both subgroups. Prior studies showed improved VO_2peak_ for healthy untrained adults [26] and people with chronic heart failure who completed an 8-week yoga training program [50]. We add to these prior studies by showing an improved VO_2max_ for apparently healthy adults assigned to a 12-week yoga training program. While the mechanisms responsible for the change cannot be directly determined in this study, the increased muscular endurance resulting from yoga practice may have achieved a better control of intercostal muscles that would subsequently improve VO_2max_.

Previous studies have reported that yoga exercise improved truck dynamic muscular strength and endurance as well as abdominal muscles muscular strength [27, 29]. Similar data were observed with a significant improvement in lower limb muscular endurance in older adults [28]. Our study also demonstrated significant favorable effects of the yoga intervention on muscular strength and endurance of upper body muscles and abdominal muscles in both men and women. The mechanisms by which yoga provides beneficial effects on muscular strength and endurance still need to be determined but it is reasonable to believe that the benefit may be mediated by some yoga postures, which may have achieved optimal intensity for increasing muscular strength and endurance. A previous electromyographic (EMG) analysis in 20 older adults showed that some selected yoga postures including the* Chair* and* Warrior Front* postures had generated relatively large knee extensor joint moments of force and quadriceps EMG activity [51]. In addition, yoga may help to maintain proper posture and spinal alignment, thereby exerting beneficial effects on muscular strength and endurance. However, abdominal muscular strength failed to improve in the yoga group versus the control group within older participants. It is possible that the intensity of yoga was not sufficiently high to provide observable improvements in this measure in older adults who tend to have weak abdominal muscles. However, we did not measure muscle mass in this study.

An additional benefit of the yoga intervention was improvement in flexibility, which can determine the efficiency of muscles. Our findings demonstrated significant favorable effects of the yoga program on lower back and hamstring flexibility in both genders and age groups. These results are in accordance with previous research that found yoga significantly improved ankle flexibility, shoulder elevation, trunk extension, and trunk flexion in healthy untrained adults [26] and spinal flexibility in people with chronic low back pain [52]. The findings of the increased flexibility are not unexpected, given the static stretching nature of yoga postures (involving holding the stretched position using the strength of the agonist muscle) [26], which exert their beneficial effects on flexibility by increasing the length of both connective and muscle tissue [53], thus engendering a range of joint angles.

The feasibility of the yoga program was also demonstrated by (1) the low attrition rate (11.0%), which was lower compared to overseas studies with similar study design (15.3–22.0%) [54, 55], (2) the good attendance, with participants attending an average of 94% of available sessions, and (3) the positive feedback from participants, with all of them suggesting that the intervention was well received and most of them believing yoga is helpful for enhancing their health. Compliance was good, with most of the participants reported practicing yoga at home. Although most of the participants reported some difficulties with some of the yoga postures at the beginning of the intervention, class attendance was high. Therefore, our results suggest that Hatha yoga may be an alternative training modality for health enhancements.

The limitations of this study include the lack of randomization, which may have led to selection bias and decreased comparability between groups with various confounding factors. Other limitations include nonblinded assessment of outcomes and the possible lack of measures of confounding factors such as diet and sleep quality. In addition, the participants were a highly motivated group that was willing to volunteer for a research study, and the yoga class was adapted for beginners; therefore findings may not be directly generalizable to a typical community yoga class. Finally, because the yoga classes were offered to participants once per week, the frequency of sessions may be different from that of other intervention studies, which makes comparison between studies difficult. However, our study has several notable strengths including the use of an expert yoga practitioner to design a program specifically for middle-aged to older adults, a sex-balanced stratified sample, and the inclusion of multiple outcome variables.

In conclusion, this study showed that a 12-week Hatha yoga program produced beneficial changes in cardiovascular endurance, muscular strength and endurance, and flexibility for Chinese adults. Given the high adherence and attendance rates as well as the encouraging results of this study, yoga may be a promising alternative form of exercise. Further investigation with longer follow-up (e.g., 6 months) should be considered, which would offer insights as to the long-term benefits of yoga.

## Figures and Tables

**Table 1:** Demographic characteristics of participants, by gender (*N* = 173).

Characteristics	Men (*n* = 64)	Women (*n* = 109)	*P*
	M ± SD or *f* (%)		
Age, years	53.59 ± 8.02	51.04 ± 6.97	0.029
Marital status, %^†^			
Single	2 (3.1)	25 (22.9)	0.050
Married	57 (89.1)	84 (77.1)	
Widowed	5 (7.8)	0 (0)	
Education level, %^†^			<0.001
No education	0 (0)	1 (0.9)	
Primary	0 (0)	10 (9.2)	
Secondary	29 (45.3)	70 (64.2)	
Tertiary	35 (54.7)	28 (25.7)	
Occupation, %^†^			0.973
Full-time	37 (57.8)	56 (51.4)	
Part-time	1 (1.6)	9 (8.3)	
Unemployed	2 (3.1)	0 (0)	
Housewife	0 (0)	30 (27.5)	
Retired	24 (37.5)	14 (12.8)	
Medication use, %			
Antidiabetic			0.489
Yes	10 (15.6)	13 (11.9)	
No	54 (84.4)	96 (88.1)	
Antihypertensive			0.118
Yes	14 (21.9)	36 (33.0)	
No	50 (78.1)	73 (67.0)	
Lipid lowering			0.146
Yes	6 (9.4)	19 (17.4)	
No	58 (90.6)	90 (82.6)	
Smoking, %^†^			<0.001
Current	3 (4.7)	0 (0)	
Quitted	10 (15.6)	1 (0.9)	
Never	51 (79.7)	108 (99.1)	
Alcohol intake, %^†^			0.004
Quitted	2 (3.1)	0 (0)	
Never	18 (28.1)	55 (50.5)	
Sometimes	40 (62.5)	52 (47.7)	
Always	4 (6.3)	2 (1.8)	
Physical activity level, MET-minutes/week	1450.86 ± 918.85	1936.63 ± 1926.52	0.041
HRQoL			
Physical function (PF)	91.09 ± 10.96	85.00 ± 11.30	0.001
Role physical (RP)	88.67 ± 23.12	81.64 ± 29.59	0.085
Bodily pain (BP)	70.55 ± 21.35	64.52 ± 20.70	0.065
General health perceptions (GH)	60.16 ± 11.41	61.24 ± 12.25	0.566
Physical component score (PCS)	310.47 ± 47.30	292.31 ± 56.03	0.031
Vitality (VT)	69.14 ± 13.99	64.45 ± 16.82	0.050
Social functioning (SF)	87.89 ± 14.26	85.21 ± 18.72	0.290
Role emotional (RE)	85.42 ± 29.02	78.59 ± 34.10	0.164
General mental Health (MH)	78.06 ± 12.75	75.71 ± 14.74	0.288
Mental component score (MCS)	320.51 ± 53.10	303.96 ± 70.87	0.083
Body measurements			
SBP, mmHg	132.52 ± 18.21	129.11 ± 18.69	0.244
DBP, mmHg	79.56 ± 11.01	78.57 ± 11.41	0.576
BMI, kg/m^2^	24.83 ± 3.65	25.37 ± 4.08	0.388
Outcome measures			
Cardiorespiratory endurance			
Resting heart rate, bpm	67.17 ± 9.14	69.88 ± 9.53	0.069
VO_2max_, mL/kg/min	32.97 ± 6.71	25.50 ± 5.01	<0.001
Muscular strength and endurance			
Curl-ups, times	21.25 ± 5.50	20.18 ± 5.19	0.204
Push-ups, times	8.17 ± 8.09	1.84 ± 3.83	<0.001
Flexibility			
MBS left leg, cm	44.34 ± 13.15	51.85 ± 9.62	<0.001
MBS right leg, cm	44.30 ± 13.71	51.63 ± 9.89	<0.001

The total percentage may not add up exactly to 100% due to rounding.

BMI: body mass index; DBP: diastolic blood pressure; HRQoL: health-related quality of life; MBS: the modified back-saver sit-and-reach test; SBP: systolic blood pressure; VO_2max_: maximal oxygen uptake.

^†^Variables were regrouped for analyses as follows: marital status (single + widowed versus married), education level (No education + primary + secondary versus tertiary), occupation (full-time + part-time versus unemployed + housewife + retired), smoking (current + quitted versus never), and alcohol intake (quitted + sometimes + always versus never).

**Table 2:** Demographic characteristics of participants in the yoga group and the control group, by gender.

Characteristics	Men			Women		
	Yoga (*n* = 34)	Control (*n* = 30)	*P*	Yoga (*n* = 53)	Control (*n* = 56)	*P*
	M ± SD or *f* (%)			M ± SD or *f* (%)		
Age, years	53.68 ± 7.91	53.50 ± 8.27	0.931	51.64 ± 6.57	50.46 ± 7.35	0.381
Marital status, %^†^			0.067			0.589
Single	2 (5.9)	0 (0)		11 (20.8)	14 (25.0)	
Married	28 (82.4)	29 (96.7)		42 (79.2)	42 (75.0)	
Widowed	4 (11.8)	1 (3.3)		0 (0.0)	0 (0)	
Education level, %^†^			0.087			0.543
No education	0 (0)	0 (0)		0 (0)	1 (1.8)	
Primary	0 (0)	0 (0)		5 (9.4)	5 (8.9)	
Secondary	12 (35.3)	17 (56.7)		33 (62.3)	37 (66.1)	
Tertiary	22 (64.7)	13 (43.3)		15 (28.3)	13 (23.2)	
Occupation, %^†^			0.355			0.813
Full-time	21 (61.8)	16 (53.5)		27 (50.9)	29 (51.8)	
Part-time	1 (2.9)	0 (0)		4 (7.5)	5 (8.9)	
Unemployed	0 (0)	2 (6.7)		0 (0)	0 (0)	
Housewife	0 (0)	0 (0)		18 (34.0)	12 (21.4)	
Retired	12 (35.3)	12 (40)		4 (7.5)	10 (17.9)	
Medication use, %						
Antidiabetic			0.064			0.688
Yes	8 (23.5)	2 (6.7)		7 (13.2)	6 (10.7)	
No	26 (76.5)	28 (93.3)		46 (86.8)	50 (89.3)	
Antihypertensive			0.791			0.066
Yes	7 (20.6)	7 (23.3)		13 (24.5)	23 (41.1)	
No	27 (79.4)	23 (76.7)		40 (75.5)	33 (58.9)	
Lipid lowering			0.872			0.904
Yes	3 (8.8)	3 (10.0)		9 (17.0)	10 (17.9)	
No	31 (91.2)	27 (90.0)		44 (83.0)	46 (82.1)	
Smoking, %^†^			0.953			0.328
Current	1 (2.9)	2 (6.7)		0 (0)	1 (1.8)	
Quitted	6 (17.6)	4 (13.3)		0 (0)	0 (0)	
Never	27 (79.4)	24 (80.0)		53 (100)	55 (98.2)	
Alcohol intake, %^†^			0.384			0.069
Quitted	1 (2.9)	1 (3.3)		0 (0)	0 (0)	
Never	8 (23.5)	10 (33.3)		22 (41.5)	33 (58.9)	
Sometimes	23 (67.6)	17 (56.7)		30 (56.6)	22 (39.3)	
Always	2 (5.9)	2 (6.7)		1 (1.9)	1 (1.8)	
Physical activity level, MET-minutes/week	1332.36 ± 1047.76	1569.36 ± 769.31	0.330	2109.24 ± 2145.74	1781.99 ± 1714.95	0.422
HRQoL						
Physical function (PF)	93.24 ± 5.89	88.67 ± 14.50	0.096	85.85 ± 11.88	84.20 ± 10.78	0.448
Role physical (RP)	91.18 ± 19.35	85.83 ± 26.82	0.360	77.36 ± 32.62	85.71 ± 26.05	0.141
Bodily pain (BP)	71.26 ± 19.07	69.73 ± 23.99	0.777	62.57 ± 19.87	66.18 ± 21.49	0.365
General health perceptions (GH)	61.03 ± 11.33	59.17 ± 11.60	0.519	60.47 ± 12.37	61.96 ± 12.20	0.527
Physical component score (PCS)	316.71 ± 33.06	303.40 ± 59.33	0.282	286.25 ± 60.58	298.05 ± 51.23	0.273
Vitality (VT)	71.03 ± 12.30	67.00 ± 15.62	0.253	64.06 ± 16.11	64.82 ± 17.61	0.814
Social functioning (SF)	86.40 ± 13.89	89.58 ± 14.71	0.376	83.25 ± 20.35	87.05 ± 17.02	0.292
Role emotional (RE)	80.39 ± 32.94	91.11 ± 23.05	0.133	71.07 ± 37.00	85.71 ± 29.72	0.025
General mental Health (MH)	77.65 ± 11.49	78.53 ± 14.23	0.784	73.74 ± 15.08	77.57 ± 14.30	0.176
Mental component score (MCS)	315.47 ± 53.26	326.23 ± 53.23	0.423	292.12 ± 75.16	315.16 ± 65.25	0.090
Body measurements						
SBP, mmHg	132.82 ± 16.80	132.17 ± 19.97	0.887	125.87 ± 15.21	132.18 ± 21.16	0.076
DBP, mmHg	78.32 ± 10.79	80.97 ± 11.27	0.342	76.64 ± 10.43	80.39 ± 12.07	0.086
BMI, kg/m^2^	24.28 ± 4.21	25.45 ± 2.83	0.203	24.55 ± 3.62	26.14 ± 4.37	0.041
Outcome measures						
Cardiorespiratory endurance						
Resting heart rate, bpm	67.53 ± 9.31	66.77 ± 9.10	0.742	69.11 ± 9.06	70.61 ± 9.97	0.416
VO_2max_, mL/kg/min	32.99 ± 6.77	32.95 ± 6.76	0.982	26.35 ± 5.51	24.69 ± 4.37	0.083
Muscular strength and endurance						
Curl-ups, times	20.18 ± 6.42	22.47 ± 4.00	0.088	21.04 ± 5.43	19.38 ± 4.86	0.095
Push-ups, times	9.50 ± 9.50	6.67 ± 5.93	0.153	1.87 ± 4.30	1.82 ± 3.37	0.950
Flexibility						
MBS left leg, cm	39.99 ± 11.47	49.28 ± 13.36	0.004	52.46 ± 8.44	51.28 ± 10.66	0.520
MBS right leg, cm	39.66 ± 11.80	49.56 ± 14.00	0.003	52.41 ± 8.31	50.89 ± 11.21	0.424

The total percentage may not add up exactly to 100% due to rounding.

BMI: body mass index; DBP: diastolic blood pressure; HRQoL: health-related quality of life; MBS: the modified back-saver sit-and-reach test; SBP: systolic blood pressure; VO_2max_: maximal oxygen uptake.

^†^Variables were regrouped for analyses as follows: marital status (single + widowed versus married), education level (No education + primary + secondary versus tertiary), occupation (full-time + part-time versus unemployed + housewife + retired), smoking (current + quitted versus never), and alcohol intake (quitted + sometimes + always versus never).

**Table 3:** Comparison of changes of outcome variables among participants in the yoga group and the control group, by gender.

Outcome variables	Yoga			Control			ANOVA		ANCOVA	
	Pre	Post	Post-Pre	Pre	Post	Post-Pre	*P*	Effect size	Adjusted	Effect size
		M ± SD			M ± SD			partial *η* ^2^	*P*	partial *η* ^2^
Men	*n* = 34			*n* = 30						
Resting heart rate, bpm	67.53 ± 9.31	66.32 ± 9.79	−1.21 ± 6.07	66.77 ± 9.10	68.73 ± 9.77	1.97 ± 8.41	0.086	0.047	/	/
VO_2max_, mL/kg/min	32.99 ± 6.77	35.59 ± 6.63	2.61 ± 2.56	32.95 ± 6.76	33.66 ± 6.18	0.72 ± 2.84	0.007	0.113	/	/
Curl-ups, times	20.18 ± 6.42	22.59 ± 5.28	2.41 ± 4.40	22.47 ± 4.00	23.13 ± 3.18	0.67 ± 2.17	0.046	0.059	/	/
Push-ups, times	9.50 ± 9.50	13.12 ± 10.26	3.62 ± 3.07	6.67 ± 5.93	7.27 ± 6.21	0.60 ± 2.27	<0.001	0.239	/	/
MBS left leg, cm	39.99 ± 11.47	46.09 ± 11.20	6.10 ± 5.11	49.28 ± 13.36	49.00 ± 13.46	−0.28 ± 3.29	<0.001	0.356	/	/
MBS right leg, cm	39.66 ± 11.80	46.06 ± 11.22	6.40 ± 5.04	49.56 ± 14.00	49.22 ± 13.47	−0.34 ± 4.00	<0.001	0.357	/	/

Women	*n* = 53			*n* = 56						
Resting heart rate, bpm	69.11 ± 9.06	66.81 ± 7.17	−2.30 ± 6.90	70.61 ± 9.97	71.39 ± 11.07	0.79 ± 7.00	0.022	0.048	/	/
VO_2max_, mL/kg/min^†^	26.35 ± 5.51	27.68 ± 5.25	1.33 ± 2.65	24.69 ± 4.37	24.27 ± 4.79	−0.42 ± 2.66	0.001	0.100	0.002	0.087
Curl-ups, times	21.04 ± 5.43	23.92 ± 3.27	2.89 ± 4.51	19.38 ± 4.86	20.04 ± 5.46	0.66 ± 4.66	0.013	0.057	/	/
Push-ups, times	1.87 ± 4.30	5.28 ± 5.62	3.42 ± 2.56	1.82 ± 3.37	1.82 ± 2.98	0.00 ± 1.85	<0.001	0.375	/	/
MBS left leg, cm	52.46 ± 8.44	58.66 ± 7.49	6.20 ± 4.19	51.28 ± 10.66	50.82 ± 10.96	−0.46 ± 4.07	<0.001	0.397	/	/
MBS right leg, cm	52.41 ± 8.31	58.95 ± 7.40	6.55 ± 4.22	50.89 ± 11.21	50.87 ± 11.71	−0.03 ± 4.36	<0.001	0.374	/	/

Pre: baseline; Post: postintervention; Post-Pre: change from baseline to postintervention; *η* ^2^: eta-squared; VO_2max_: maximal oxygen uptake; MBS: the modified back-saver sit-and-reach test.

The effect size in ANOVA/ANCOVA is represented by value of partial eta-squared (*η* ^2^) as very small (<0.01), small (0.01–0.05), medium (0.06–0.13), and large (≥0.14).

^†^Adjusted for BMI at baseline and HRQoL role emotional domain at baseline.

**Table 4:** Comparison of changes of outcome variables among participants in the yoga group and the control group, by age group.

Outcome variables	Yoga			Control			ANOVA		ANCOVA	
	Pre	Post	Post-Pre	Pre	Post	Post-Pre	*P*	Effect size	Adjusted	Effect size
		M ± SD			M ± SD			partial η^2^	*P*	partial η^2^
<53 years	*n* = 40			*n* = 45						
Resting heart rate, bpm	68.93 ± 9.24	67.25 ± 7.01	−1.68 ± 5.47	69.82 ± 10.62	71.64 ± 11.39	1.82 ± 6.88	0.012	0.074	/	/
VO_2max_, mL/kg/min	30.31 ± 6.75	31.71 ± 7.29	1.40 ± 2.45	28.18 ± 6.76	27.79 ± 7.15	−0.39 ± 2.99	0.004	0.098	/	/
Curl-ups, times	19.95 ± 6.50	23.00 ± 4.71	3.05 ± 4.79	20.64 ± 4.27	21.00 ± 5.01	0.36 ± 4.13	0.007	0.085	/	/
Push-ups, times	4.90 ± 7.80	7.95 ± 9.25	3.05 ± 2.53	3.51 ± 5.06	4.09 ± 4.57	0.58 ± 1.96	<0.001	0.236	/	/
MBS left leg, cm	47.75 ± 9.97	54.44 ± 9.10	6.69 ± 5.19	49.67 ± 10.21	49.37 ± 10.90	−0.30 ± 4.39	<0.001	0.352	/	/
MBS right leg, cm	48.09 ± 9.83	54.83 ± 8.99	6.74 ± 4.71	49.03 ± 10.57	49.14 ± 11.42	0.12 ± 4.69	<0.001	0.336	/	/

≥53 years	*n* = 47			*n* = 41						
Resting heart rate, bpm^†^	68.13 ± 9.13	66.09 ± 9.20	−2.04 ± 7.45	68.66 ± 8.89	69.17 ± 9.76	0.51 ± 8.14	0.128	0.027	0.125	0.080
VO_2max_, mL/kg/min^†^	27.78 ± 6.74	29.98 ± 6.67	2.20 ± 2.83	26.90 ± 6.44	27.28 ± 6.78	0.38 ± 2.45	0.002	0.106	0.002	0.105
Curl-ups, times	21.34 ± 5.16	23.74 ± 3.73	2.40 ± 4.17	20.24 ± 5.35	21.24 ± 5.04	1.00 ± 3.77	0.103	0.031	/	/
Push-ups, times^†^	4.81 ± 7.76	8.68 ± 8.12	3.87 ± 2.91	3.51 ± 4.95	3.32 ± 4.62	−0.20 ± 2.02	<0.001	0.396	<0.001	0.390
MBS left leg, cm^†^	47.45 ± 12.68	53.16 ± 12.39	5.71 ± 3.91	51.59 ± 13.07	51.09 ± 12.88	−0.50 ± 3.07	<0.001	0.438	/	/
MBS right leg, cm	46.86 ± 12.98	53.14 ± 12.54	6.28 ± 4.42	51.96 ± 13.73	51.55 ± 13.22	−0.42 ± 3.37	<0.001	0.406	<0.001	0.438

Pre: baseline; Post: postintervention; Post-Pre: change from baseline to postintervention; η^2^: eta-squared; VO_2max_: maximal oxygen uptake; MBS: the modified back-saver sit-and-reach test.

The effect size in ANOVA/ANCOVA is represented by value of partial eta-squared (*η* ^2^) as very small (<0.01), small (0.01–0.05), medium (0.06–0.13), and large (≥0.14).

^†^Resting heart rate/push-up, adjusted for DBP at baseline; VO_2max_, adjusted for BMI at baseline; left leg flexibility, adjusted for HRQoL MCS domain score at baseline.
